# Seasonal Dimorphism in the Compound Eye Morphology of *Scythris sinensis* (Felder & Rogenhofer, 1875) (Lepidoptera: Scythrididae)

**DOI:** 10.3390/insects17070702

**Published:** 2026-07-07

**Authors:** Haifeng Zhou, Yu Liang, Qing Zhang, Kang Lou

**Affiliations:** College of Life Sciences, Zhengzhou University, Zhengzhou 450001, China

**Keywords:** ommatidia, compound eye, seasonal dimorphism, ultrastructure, Lepidoptera

## Abstract

*Scythris sinensis* (Felder & Rogenhofer) is a diurnal moth characterized by its distinct spring and autumn forms. In our study, we analyzed the compound eye morphology of both forms utilizing scanning electron microscopy. The eyes are ellipsoidal and symmetrical, consisting of hexagonal ommatidia characterized by convoluted folds but lacking corneal nipples. We found that female moths have slightly larger compound eyes than males. Our counts of ommatidia indicate that males emerging in autumn (589–675) typically possess a higher number than males emerging in spring (492–698). This finding suggests an adaptation specific to the autumn period, potentially enhancing visual search efficiency in response to seasonal light variations and a constrained reproductive schedule. This research provides a foundational understanding of the visual behavior of *S*. *sinensis* concerning photoreception and phototaxis.

## Simple Summary

Visual environments vary among insect species according to their daily activity patterns and seasonal conditions, creating different selective pressures on the visual system. Our research focused on the daytime moth species *Scythris sinensis* (Felder & Rogenhofer, 1875), which displays distinct forms in spring and autumn. By employing scanning electron microscopy, we sought to determine whether the structure of the compound eyes differ between these seasonal forms. Our results indicated that while female moths tend to have slightly larger eyes overall, male moths captured in autumn possess a greater number of individual light-sensing units compared to their spring counterparts. This finding implies that autumn males may have developed slightly improved vision to aid in mate detection during the shorter days and specific light conditions of fall. This study enhances our understanding of how insects adapt their sensory systems to survive and reproduce in an evolving environment.

## 1. Introduction

**Figure 1:**
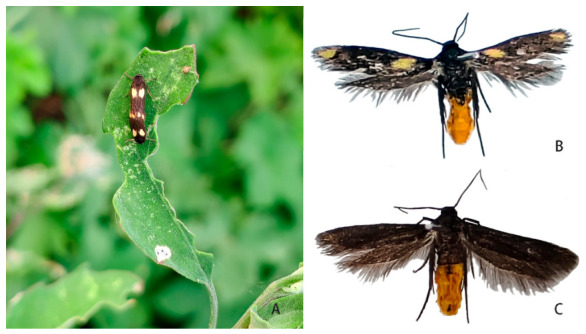
Adults of *S*. *sinensis*. (**A**) Mating pair of the autumn form on a *C. album* host leaf; (**B**) Autumn form; (**C**) Spring form.

Seasonal polyphenism is relatively common among Lepidoptera, often evident in variations in body color and wing patterns [1,2,3]. As a diurnal micro-moth, *Scythris sinensis* (Felder & Rogenhofer) exhibits seasonal polyphenism similar to that found in some butterflies (Figure 1A). *S. sinensis* produces two to three generations annually [4]. Adults active in the spring typically feature entirely black forewings, whereas the second generation, which is generally active from summer to autumn, exhibits yellow spots at both the base one-third and the apex of the forewings (Figure 1B,C) [5]. Its larvae are gregarious during the first two instars and feed specifically on plants of the genus *Chenopodium*, notably *C. album* Linnaeus, a common agricultural weed [6,7]. This relatively specialized feeding habit allows *S. sinensis* to reduce the reproductive base of *C. album* in the following year, thereby contributing to the control of this weed’s proliferation [8].

The compound eye is the principal visual organ of insects and is composed of numerous ommatidia, whose number and morphology vary considerably among insect orders and ecological contexts. The number of ommatidia is closely associated with visual performance, particularly spatial resolution and light sensitivity, and ranges from only a few dozen in species with reduced visual demands to many thousands in visually specialized taxa such as Odonata, Diptera, Hymenoptera, and Lepidoptera [9]. Across species, the number of ommatidia varies from as few as 29 in certain wasps to tens of thousands in dragonflies [10,11]. In many insects, males possess larger compound eyes, a greater number of ommatidia, or specialized eye regions that enhance mate detection and flight-related visual performance [12,13,14,15]. Substantial intraspecific variation has also been reported; for example, different strains of *Drosophila melanogaster* may differ by several hundred ommatidia [14,16,17,18]. In addition to genetic, interspecific, and sex-related variation, compound-eye morphology can exhibit phenotypic plasticity in response to seasonal and environmental conditions. Factors such as photoperiod, developmental temperature, nutrition, and body size have all been shown to influence eye development and ultimately affect the number of ommatidia [19,20].

Despite extensive documentation of differences in compound-eye morphology among species and between sexes, relatively little is known about variation in the number of ommatidia in seasonally polyphenic insects. The striking contrast in forewing patterns between the spring and autumn forms of *S*. *sinensis* offers an excellent model for investigating whether seasonal polyphenism is also associated with changes in compound-eye morphology, particularly in ommatidia number [21,22].

By comparing the ommatidia number in the compound eyes of spring and autumn forms of *S*. *sinensis* using scanning electron microscopy (SEM), this study examines whether seasonal polyphenism is associated with differences in compound-eye morphology.

## 2. Materials and Methods

### 2.1. Insect Collection

The primary host plant of *S*. *sinensis* is *C. album*. Adults were netted on *C*. *album* along a roadside in Fengzhuang Village (34.87°, 113.48°), Guangwu Town, Zhengzhou City, in proximity to Zhengzhou University.

### 2.2. Scanning Electron Microscopy (SEM)

The samples were rinsed three times for 15 min each in a 0.1 molar phosphate buffer (pH 7.2), followed by 30 min fixation in a fixative solution consisting of 2.5% glutaraldehyde in 0.1 molar phosphate buffer (pH 7.2) at 4 °C. Dehydration was performed using a graded ethanol series, including concentrations of 75%, 80%, 90%, and 95%, with each concentration lasting 10 min. This was followed by two changes of 100% ethanol, each for 20 min. After drying with a critical point dryer (Quorum K850, Quorum Technologies, Lewes, UK), the samples were gold-coated for approximately 30 s using an ion sputter coater (Hitachi MC1000, Tokyo, Japan) and then examined with a Hitachi SU8100 field emission scanning electron microscope (Hitachi, Tokyo, Japan) at a 3 kV accelerating voltage.

### 2.3. Data Measurement and Analysis

A total of 32 compound eye samples were used in this study for morphological and anatomical analysis, comprising 6 spring females, 10 spring males, 8 autumn females, and 8 autumn males. The sample size for specific measurements is indicated by “n” in the text. All measurements were conducted using ImageJ software (Version 1.50i). The structural parameters of the compound eyes were quantified at two levels using SEM images: (1) the gross morphology of the entire compound eye, including the projected area of the compound eye and the total number of ommatidia, and (2) the fine morphology of individual corneal facets, including facet diameter, facet area, facet perimeter, and inter-facet spacing. The projected area of each compound eye was obtained by manually outlining the eye boundary in ImageJ after scale calibration, followed by automatic area calculation using the Measure function. For facet-level measurements, a standardized central region of the compound eye was selected in each specimen to ensure consistency among individuals and species. Facet area and perimeter were measured in ImageJ after outlining the boundary of each facet. Facet diameter was defined as the diagonal distance across the hexagonal facet, and inter-facet spacing was measured as the distance between the centers of two adjacent facets. All statistical analyses and visualizations were performed using R (version 4.3.2) within the RStudio environment (version 2025.09.2). Prior to comparative analysis, the normality of data distribution for each group was assessed using the Shapiro–Wilk test, while homogeneity of variances was evaluated with F-tests. The primary comparisons of interest (a, spring female vs. male; b, autumn female vs. male; c, spring vs. autumn female; d, spring vs. autumn male) were conducted using independent samples *t*-tests. When the assumption of equal variances was satisfied (*p* > 0.05 in the F-test), a standard Student’s *t*-test was applied; otherwise, Welch’s correction was used. The significance threshold (α) was set at 0.05 for all tests.

## 3. Results

**Figure 2:**
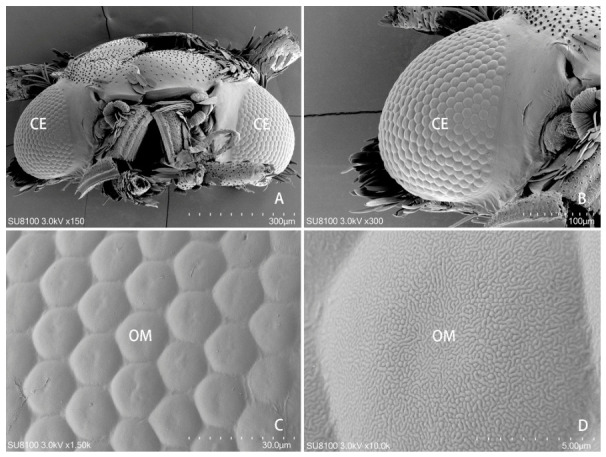
External morphology of compound eyes in spring female *S*. *sinensis*, SEM. (**A**) Frontal view of the head. (**B**) Left compound eye. (**C**) Ommatidia. (**D**) Convoluted folds on the ommatidial facets. CE, compound eye; OM, ommatidium.

The adults of the spring and autumn forms of *S*. *sinensis* possess compound eyes that are symmetrically located at the bases of the antennae on the head (Figure 2A,B). Viewed from the frontal perspective, the compound eyes are positioned on either side of the head, with the left and right eyes exhibiting largely identical external morphology. They have an ellipsoidal shape and a glossy appearance. The surfaces of facets are slightly convex and arranged in a regular, tight, and orderly pattern. Most facets are hexagonal, with occasional pentagonal shapes; their outer surfaces lack corneal nipples but display convoluted folds reminiscent of the cortex pattern found in primate brains (Figure 2C,D).

Independent-samples *t*-test showed significant differences in both area and the perimeter of the corneal facets between females and males of each form (spring and autumn). However, no significant differences were detected when comparing the spring and autumn forms as a whole (Table 1).

Regarding facet diameter, a significant difference was found between females of the spring and autumn forms, while all measured parameters exhibited significant differences between the males of the two forms. In terms of the projected area of compound eye and individual facet area, females of both forms showed slightly larger sizes than their male counterparts. Nevertheless, no significant differences were identified among any groups based on sex or form specifically for the projected area of compound eye.

**Figure 3:**
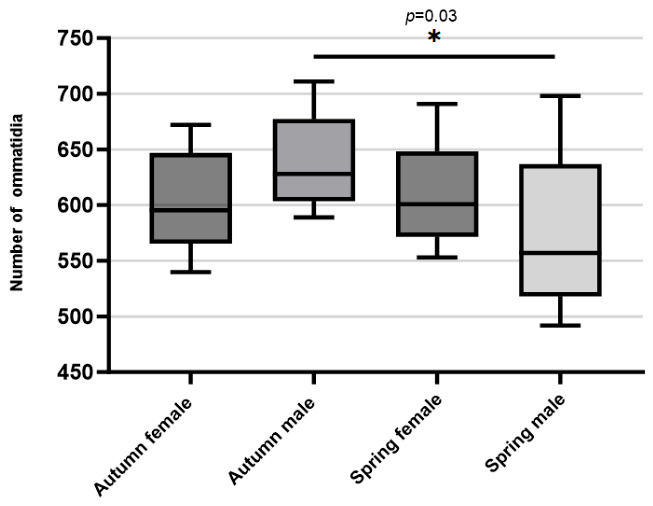
Ommatidia counts in *S*. *sinensis*. Data are shown as mean ± SEM with individual points (n as indicated). * *p* < 0.05 (*t*-test).

The mean number of ommatidia (±SEM) was 609.67 ± 19.8 in spring-emerged females, 575.0 ± 21.8 in spring-emerged males, 602.5 ± 16.42 in autumn-emerged females, and 640.25 ± 15.21 in autumn-emerged males (Figure 3). The number of ommatidia was compared across seasons and sexes using independent samples *t*-tests. A significant seasonal difference was identified specifically between males, with autumn males exhibiting a significantly higher number of ommatidia compared to spring males (t = −2.334, df = 16, *p* = 0.033, Cohen’s d = 1.107, statistical power 0.71). In contrast, no significant difference was found between spring and autumn females (t = 0.280, df = 12, *p* = 0.784, Cohen’s d = 0.151, statistical power 0.09). When data were pooled by sex, the overall seasonal comparison (spring vs. autumn morphs) did not reach statistical significance (t = −1.698, df = 30, *p* = 0.100, Cohen’s d = 0.601, statistical power 0.42). Similarly, the overall comparison between all males and all females, regardless of season, also showed no significant difference (t = 0.076, df = 30, *p* = 0.940, Cohen’s d = 0.027, statistical power 0.05). These results suggest that variation in the number of ommatidia may be associated primarily with a season-specific increase in males. However, the absence of significant differences in the other comparisons should be interpreted cautiously given the relatively low statistical power of several tests.

## 4. Discussion

The diurnal adaptation in Lepidoptera has undergone multiple phylogenetic divergences; Kawahara et al. estimated that approximately 15% to 25% of lepidopteran species are diurnal [23]. Within this range, the majority of species in the family Scythrididae are diurnal [24]. The transition from nocturnal to diurnal habits involves various adaptations in visual structures. For instance, most nocturnal species possess superposition eyes, while diurnal species typically have apposition eyes [25]. In addition to eye type, adaptations also occur on the corneal surface in response to changes in the optical environment. In this study, we observed that *S*. *sinensis* lacks distinct corneal nipples on the ommatidia, a feature present in other diurnal moths such as *Euclidia mi* (Clerck), *Alcides zodiaca* Butler and *Histia flabellicornis* (Fabricius) [26,27]. The absence of nipples in *S*. *sinensis* may indicate a more advanced adaptation to diurnal life, similar to the reduction or loss of nipples observed in butterflies [28]. Furthermore, our findings support the conclusion of Spalding et al. that the appearance and disappearance of corneal nipples in Lepidoptera have undergone multiple independent evolutionary events, rather than being a trait exclusively reduced in butterflies [26].

However, light adaptation is not the only factor shaping the visual system of Lepidoptera. Characteristics of potential mates, such as wing pattern, size, and shape, also impose important selective pressures on visual performance and mating behaviors [29]. Sexual dimorphism in eye structure is a well-documented consequence of such selection. For example, in many Diptera, males often possess holoptic eyes that facilitate mate detection, whereas females typically exhibit a dichoptic eye configuration. These structural differences are frequently associated with differences in the number of ommatidia between the sexes, a trait functionally linked to the male’s role in locating potential mates [30]. Vision is the primary sensory modality in most animals and plays a central role in information acquisition and behavioral decision-making [31]. Consequently, organisms have evolved diverse visual adaptations to meet the demands of ecologically important tasks such as mate detection and visually guided flight [32,33,34]. In the present study, the number of ommatidia differed between spring-emerged males and spring-emerged females, as well as between autumn-emerged males and autumn-emerged females. However, these differences were not statistically significant. In contrast, autumn-emerged males possessed significantly more ommatidia than spring-emerged males. This pattern is consistent with the hypothesis that seasonal factors may contribute more strongly than sexual dimorphism to variation in the number of ommatidia in *S*. *sinensis*. However, this interpretation should be treated with caution because several of the non-significant comparisons had relatively low statistical power (as low as 0.05), indicating that the absence of statistical significance may partly reflect limited sample size rather than the true absence of biological differences. Additional studies with larger sample sizes will therefore be necessary to further evaluate the relative contributions of seasonal and sex-specific factors to compound eye morphology.

Seasonal changes, accompanied by shifts in temperature, light availability, and environment conditions, create a more complex visual ecology in summer and autumn compared to spring [35,36]. In many insects exhibiting seasonal polyphenism, photoperiod and temperature are the primary abiotic factors that regulate alternative developmental trajectories [37,38]. In response to these challenges, *S. sinensis* exhibits coordinated seasonal adaptations that encompass not only wing color patterns but also the morphology of its compound eyes. Specifically, the autumn morph—characterized by distinct wing markings—has a significantly higher number of ommatidia. We hypothesize that shortening day length and decreasing temperatures experienced by the larval or pupal stages cue the development of the autumn form in *S*. *sinensis*. These external signals are presumably integrated via neuroendocrine pathways, leading to hormone-mediated changes in gene expression that modulate eye development, resulting in a higher number of ommatidia. This investment in visual infrastructure is functionally interpreted as an adaptation to improve the efficiency of visually mediated mate detection under the dimmer light and tighter time constraints of the autumn reproductive season.

This increase in the number of ommatidia in autumn-emerged males suggests a sexually dimorphic pattern of seasonal plasticity in compound eye morphology, with the seasonal increase observed only in males. Such morphological changes may confer adaptive advantages in the context of autumn reproduction [13]. In particular, the enhanced visual resolution and/or sensitivity associated with increased ommatidial number may improve mate detection and flight performance under autumnal photic conditions, including reduced light intensity and altered spectral composition [39]. Although daylight duration and light intensity can vary seasonally, the extent to which these factors directly drive the observed plasticity remains uncertain. One possible explanation for this male-specific increase is that males and females experience different ecological or behavioral demands, resulting in sex-specific developmental plasticity of the compound eye. Males may rely more heavily on visually guided mate-searching and flight activity, whereas females may allocate resources differently, resulting in reduced selective pressure for similar visual enhancement [12,14]. However, the mechanisms underlying this sexually dimorphic response remain unclear, and further studies are required to determine whether differences in visual behavior, developmental regulation, or other sex-specific selective pressures contribute to this pattern. Because body size and head capsule measurements, such as inter-ocular distance, were not systematically recorded in the present study, we could not determine whether the observed differences in compound eye size were correlated with overall body or head size [40]. This represents a limitation of the study. Future work should include measurements of body size and head capsule dimensions to evaluate whether variation in compound eye size reflects general body size differences or specific changes in visual morphology.

The role of temperature as a key environmental cue is supported by geographic variation observed in August. Specimens collected from Zhengzhou (34.87° N, 113.48° E; 0 m a.s.l.), where the average August temperature is 28.5 °C, display the yellow-spotted morph, whereas those collected from the Wuzhong Forest Farm in Ningxia (37.90° N, 106.28° E; 1138 m a.s.l.), where the average August temperature is 22.9 °C, retained the black morph. This geographic pattern is consistent with the hypothesis that temperature contributes to seasonal phenotype determination, although other environmental factors associated with geographic variation cannot be excluded. This interpretation aligns with the well-documented temperature-mediated phenotypic plasticity in the African butterfly *Bicyclus anynana* (Butler), where lower developmental temperature results in dry-season adults with small eyespots, while higher temperature leads to wet-season adults with large eyespots [41,42,43]. However, it remains unclear whether temperature influences eye development directly or indirectly through hormonal pathways, as well as how it interacts with photoperiod. Future research that combines common-garden rearing experiments with behavioral assays is necessary to establish a causal link between the observed morphological changes and their fitness advantages in mate finding. This approach will ultimately help clarify the adaptive significance of this seasonal visual strategy.

Beyond its contribution to sensory ecology, our description of seasonal visual plasticity in *S. sinensis* may have implications for applied entomology. First, knowledge of sex- and season-specific visual capabilities could guide the design of more efficient visual monitoring tools, such as optimized trap colors or patterns, for population surveillance. More significantly, given that the larvae of *S. sinensis* are gregarious and feed on common agricultural weeds (e.g., *C. album*), the species itself represents a potential candidate for native weed management. Understanding the full life history and sensory ecology of the adults, including mate-finding behavior influenced by the visual adaptations reported here, is a necessary step in assessing its utility or promoting its conservation within integrated pest and weed management frameworks. Thus, this study provides a morphological foundation for future applied research aimed at harnessing or conserving this species.

## Figures and Tables

**Table 1:** Relevant structural parameters of the compound eyes of *S*. *sinensis*.

Type	Facet Perimeter (μm)	Facet Area (μm^2^)	Facet Diameter (μm)	Inter-Facet Spacing (μm)	Projected Area of Compound Eye (mm^2^)
Spring-female	46.90 ± 0.28 a	159.40 ± 1.70 a	16.23 ± 0.11 c	0.56 ± 0.035	0.083 ± 0.0017
Spring-male	48.09 ± 0.24 a d	152.36 ± 1.67 a d	16.18 ± 0.07 d	0.59 ± 0.010 d	0.076 ± 0.0022
Autumn-female	47.79 ± 0.24 b	164.91 ± 1.47 b	16.75 ± 0.11 b c	0.53 ± 0.011 b	0.081 ± 0.0012
Autumn-male	44.48 ± 0.25 b d	146.97 ± 1.81 b d	15.80 ± 0.07 b d	0.47 ± 0.007 b d	0.080 ± 0.0011

Data are presented as mean ± SEM. Lowercase letters indicate the following specific pairwise comparisons: a: between females and males of the spring form; b: between females and males of the autumn form; c: between females of the spring and autumn forms; d: between males of the spring and autumn forms (a letter is only shown when the corresponding comparison yielded a significant result, *p* < 0.05, in independent samples *t*-test).

## Data Availability

The raw data supporting the conclusions of this article will be made available by the authors on request.
